# Supplementary material for: Regional performance variation in external validation of four prediction models for severity of COVID-19 at hospital admission: An observational multi-centre cohort study
Source: PLoS One. 2021 Aug 25;16(8):e0255748. doi: 10.1371/journal.pone.0255748 (PMC8386866; doi:10.1371/journal.pone.0255748)
Supplement: S2 Table — (DOCX) [file pone.0255748.s002.docx]

**S2 Table:** **Different methods of imputation.**

|  | Xie model | Allenbach | Zhang1 | Zhang2 |
| --- | --- | --- | --- | --- |
| Single imputation; KNN | 0.87 [0.79-0.95] | 0.81 [0.74-0.88] | 0.72 [0.62-0.82] | 0.77 [0.70-0.84] |
| Single imputation; RF | 0.86 [0.78-0.94] | 0.81 [0.74-0.88] | 0.72 [0.62-0.82] | 0.77 [0.70-0.84] |
| Multiple imputation; BR | 0.86 [0.78-0.95] | 0.81 [0.74-0.88] | 0.72 [0.62-0.82] | 0.77 [0.70-0.84] |
| Multiple imputation; GP | 0.85 [0.76-0.95] | 0.80 [0.73-0.87] | 0.72 [0.62-0.82] | 0.77 [0.70-0.84] |

KNN; K-nearest neighbor, RF; Random forrest, GP; Gaussian Process Method, BR; Bayesian Rigde.
