# Supplementary figures and images for: Regional performance variation in external validation of four prediction models for severity of COVID-19 at hospital admission: An observational multi-centre cohort study
Source: PLoS One. 2021 Aug 25;16(8):e0255748. doi: 10.1371/journal.pone.0255748 (PMC8386866; doi:10.1371/journal.pone.0255748)

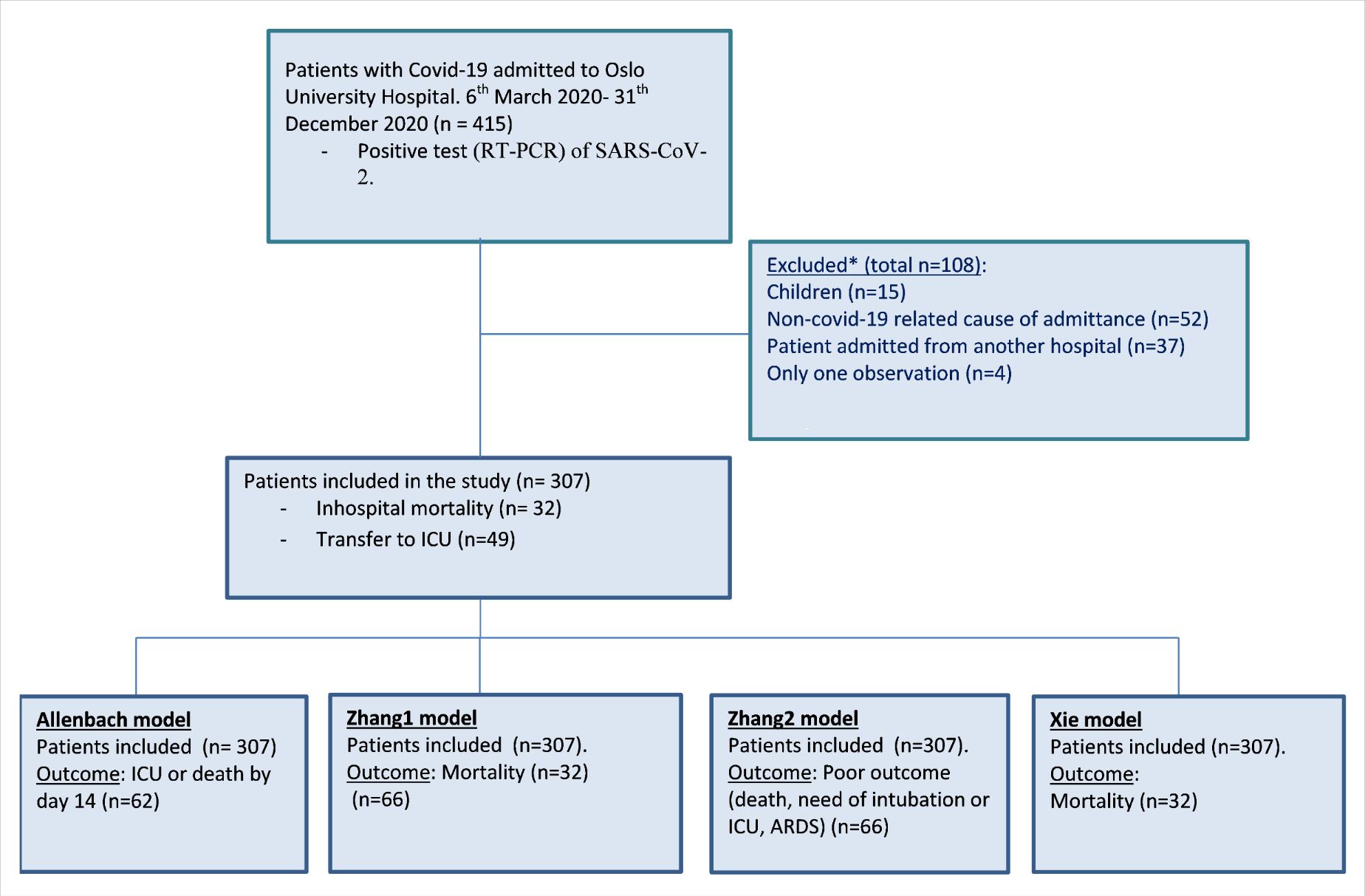

Supplement: S1 Fig — (JPEG) [file pone.0255748.s003.jpeg]

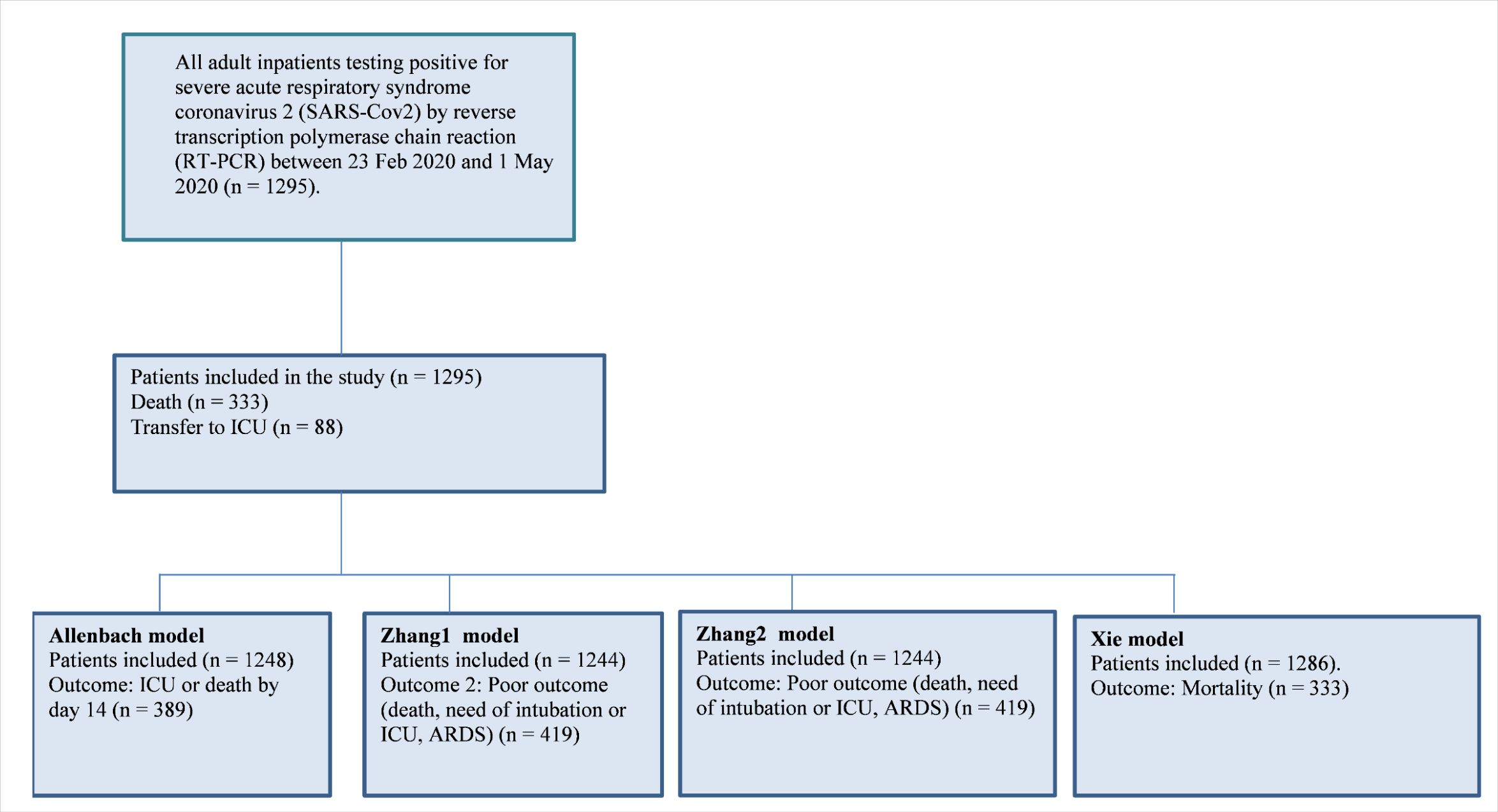

Supplement: S2 Fig — (JPEG) [file pone.0255748.s004.jpeg]

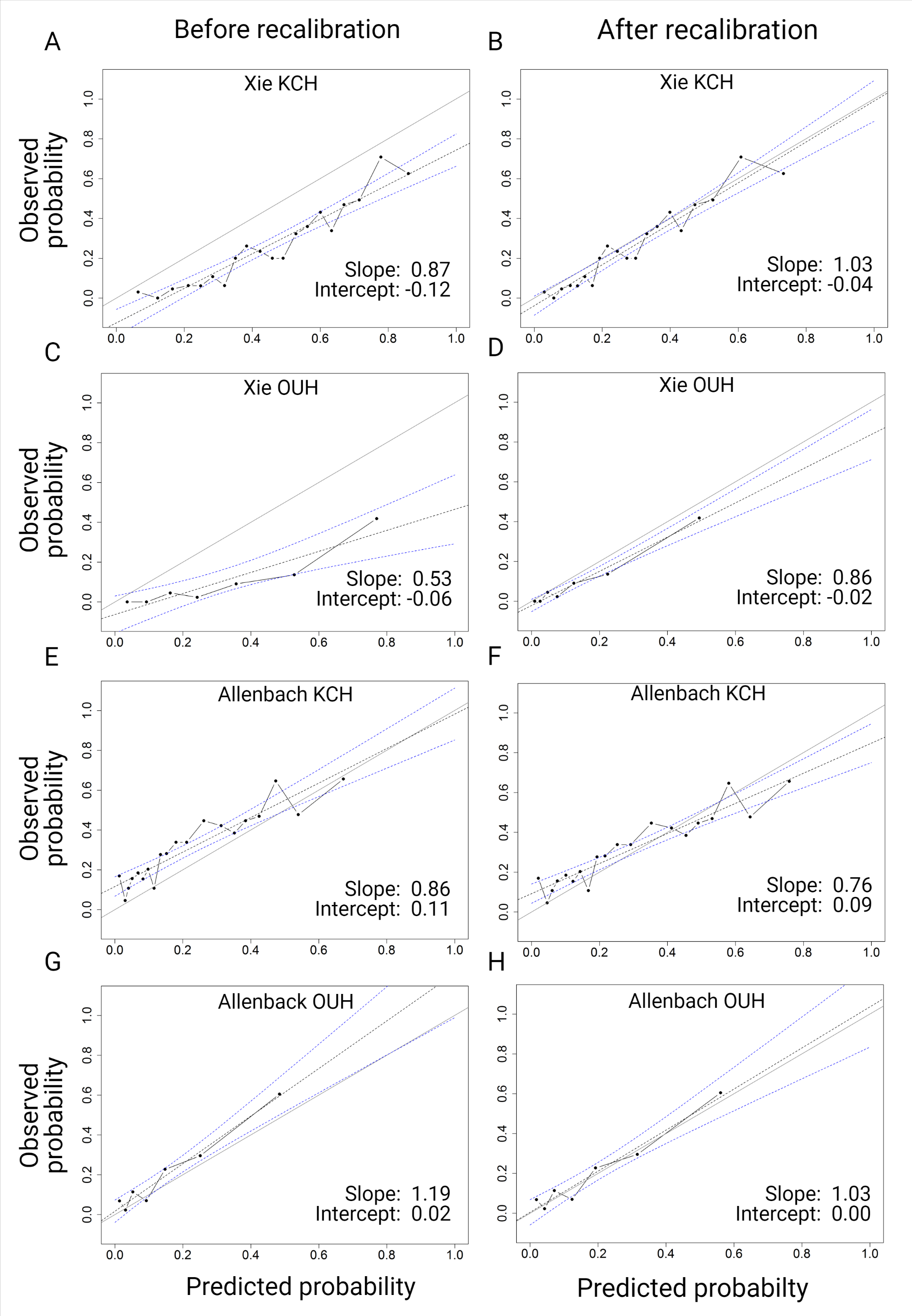

Supplement: S3 Fig — (JPEG) [file pone.0255748.s005.jpeg]
